# Paracrine Regulation of Steroidogenesis in Theca Cells by Granulosa Cells Derived from Mouse Preantral Follicles

**DOI:** 10.1155/2015/925691

**Published:** 2015-08-19

**Authors:** Xiaoqiang Liu, Pengyun Qiao, Aifang Jiang, Junyi Jiang, Haiyan Han, Li Wang, Chune Ren

**Affiliations:** ^1^Clinical Center of Reproductive Medicine, Affiliated Hospital of Weifang Medical University, Weifang, Shandong 261031, China; ^2^Key Laboratory of Reproductive Medicine of Shandong Province Health Department, Weifang, Shandong 261031, China

## Abstract

Interaction partners of follicular cells play a significant role in steroidogenesis, follicular formation, and development. Androgen secreted by theca cells (TCs) can initiate follicle development and ovulation and provide precursor materials for estrogen synthesis. Therefore, studies on ovarian microenvironment will not only lead to better understanding of the steroidogenesis but also have clinical significance for ovarian endocrine abnormalities such as hyperandrogenism in polycystic ovary syndrome (PCOS). This study applied the Transwell coculture model to investigate if the interaction between granulosa and theca cells may affect androgen production in theca cells. Concentrations of testosterone and androstenedione in the spent medium were measured by radioimmunoassay and enzyme linked immunosorbent assay, respectively. The results show that the coculture with granulosa cells (GCs) increases steroidogenesis in TCs. In addition, testosterone and androstenedione productions in response to LH stimulation were also increased in the coculture model. Significantly increased mRNA expressions of steroidogenic enzymes (*Star*, *Cyp11a1*, *Cyp17a1*, and *Hsd3b2*) were observed in the cocultured TCs. Thus, GCs were capable of promoting steroidogenesis and LH responsiveness in TCs. This study provided a basis for further exploration of ovarian endocrine mechanism and pathologies.

## 1. Introduction

Oocyte, granulosa cells (GCs), and theca cells (TCs) are the main components of follicles, and the functions and interactions among them play crucial role(s) in steroidogenesis, follicular development, and atresia [1]. Oocyte-granulosa cell communications, largely carried out by gap junctions, are essential for the female gametogenesis and formation and maturation of follicles as well as embryonic development after fertilization. Some paracrine factors such as growth differentiation factor 9 (GDF-9) and bone morphogenetic protein 15 (BMP-15) are specifically secreted by oocyte. These factors regulate follicle formation and GCs/TCs proliferation and differentiation [2, 3]. On the other hand, insulin-like growth factor-I (IGF-I), activin, and inhibin secreted by GCs are autocrine/paracrine regulators contributing to the modulation of ovarian function [4]. TCs do not only support the structure and functionality of the developing follicle but also provide androgen for estrogen biosynthesis in GCs, which stimulates the early follicle growth [5, 6].

Androgen is mainly secreted from ovary and adrenal gland. In ovaries, the steroidogenesis is accomplished by both the granulosa cells and theca cells. The synthesis of various steroids is determined by cell-specific expression of a series of steroidogenic enzymes. The transportation of cholesterol into the mitochondria is facilitated by STAR which regulates the production of androgens. It is established that the androgen-secreting theca cells express P450Scc (CYP11A1), P450C17 (CYP17), and HSD3B2, all of which are key enzymes essential for androgen biosynthesis. In hyperandrogenemia patients, the circulatory testosterone and androstenedione are primarily produced by follicular TCs of ovary [7, 8]. Although some investigator observed that oocyte-derived factors such as GDF-9 directly upregulated* Cyp17* mRNA and protein expression in rats [9, 10] and paracrine regulations of GCs may also enhance steroid production in whole follicles, many aspects of the signaling network among different groups of follicular cells, especially their effects on steroid synthesis, remain to be investigated.

*In vitro* studies have shown that, as a key feature of TCs, the increment of androgen production was driven by LH in a dose-dependent manner [5, 11, 12]. Although these findings suggested that increased LH secretion may be pivotal for the upregulation of androgen production, some other studies in women with polycystic ovary syndrome (PCOS) found that its main effect was to promote the conversion of progesterone to androgen [13]. There appears a complex network of signals between GCs and TCs on the regulation of androgen production under different pathophysiological conditions. Unfortunately, studies on GCs regulation of the steroidogenesis in TCs in early follicle development are scattered and the regulatory mechanism remains poorly understood [14]. Specifically, literature search failed to find any systematic investigation focusing on the paracrine regulation of TCs' steroidogenesis by GCs using cells derived from mouse preantral follicles.

The objective of this study is to explore the functional interactions between GCs and TCs and their involvement in steroid metabolism. We applied the Transwell coculture model to determine the effects of GCs on the steroidogenic function of TCs in the mouse preantral follicles by measuring the change of androgen concentration in response to LH stimulation. Results of the study will provide basic information on the significance of cell interactions for steroidogenesis in ovary.

## 2. Materials and Methods

### 2.1. Animals

Immature Female ICR mice for research (21 days old) were obtained from Lab Animal Center of Weifang Medical University. Mice were housed under controlled conditions with a daily lighting (12 h light and 12 h darkness). Food and water were provided* ad libitum*. All animal management procedures were carried out in accordance with the Guide for the Care and Use of Laboratory Animals and approved by the Institutional Animal Care and Use Committee.

### 2.2. Cell Isolation and Culture

TCs were isolated from mice aged 21 days and cultured following published protocols with minor modifications [15, 16]. Briefly, isolated ovaries were immediately transferred to the prewarmed Medium 199 (GIBCO, Grand Island, NY) containing 0.1% BSA, 100 IU/mL penicillin, and 100 *μ*g/mL streptomycin. Ovaries were cleaned of any surrounding tissue and repeatedly punctured with 29 G needles to release GCs. The remaining ovarian tissues were incubated in Medium 199 supplemented with 4 mg/mL collagenase I (Worthington Biochemical Corp, Lakewood, New Jersey), 10 *μ*g/mL DNase I (Worthington Biochemical Corp, Lakewood, New Jersey), and 0.1% BSA for 60 min at 37°C following three times of washing with Medium 199. Then the TCs were placed in Medium 199 containing 0.1% BSA, 1% FBS, 1% insulin-transferrin-selenium (ITS) (GIBCO, Grand Island, NY, USA), 100 IU/mL of penicillin, and 100 *μ*g/mL streptomycin. After overnight incubation, the wells were washed with Medium 199 to remove unattached cells.

For GCs collection, the released cells from the above steps were placed in prewarmed medium of DMEM/F12. Following three times of washing, the GCs were transferred to the conditioned DMEM/F12 medium containing 0.1% BSA, 10% FBS, 100 IU/mL penicillin, and 100 *μ*g/mL streptomycin. After overnight incubation, the wells were washed with DMEM/F12 medium to remove unattached cells. Cellular morphology was observed under the microscope.

### 2.3. Immunofluorescence Detection of CYP17A1 and FSHR

TCs were seeded on coverslip following washing with Medium 199 to remove unattached cells after overnight incubation. For the identification of TCs and GCs, a CYP17A1 antibody, a marker enzyme specifically expressed in TC, was used to verify the purity and identify the cells. Icy acetone was added and a coverslip was placed at −20°C for 10 minutes. After washing and permeabilization with PBS containing 0.5% Triton X-100, the cells were blocked with goat serum (Zhongshan Jinqiao Biotechnology Co. Ltd., Beijing, China) for 1 h in a humidified chamber at 37°C. Subsequently, slides were incubated with CYP17A1 antibody (1 : 200, Santa Cruz, CA) or FSHR antibody (1 : 200, Bioworld Technology, Inc., Minnesota) overnight at 4°C. Slides were incubated with secondary fluorescent FITC (1 : 100, Boster Biotechnology Co. Ltd., Wuhan, China) for 1 h in a humidified chamber at 37°C. After washing and nuclear staining with Hoechst for 10 seconds, the staining was stopped. The slides were seal-capped with Debico and observed under a fluorescence microscope.

### 2.4. Transwell Coculture of GCs and TCs

GCs were seeded in Transwell (3 *μ*m pore size, 24 mm diameter; Corning, NY) inner membrane at a density of 3 × 10^5^ and cultured with 1 mL DMEM/F12 supplemented with 0.1% BSA and antibiotics. After transfer into 6-well plate, TCs were seeded at a density of 1 × 10^5^ with Medium 199 supplemented with 0.1% BSA, 1% ITS, and antibiotics. For androgen assay and quantitative real-time PCR analyses, cells were cultured in serum-free medium for 72 h.

To assess the effect of GCs on LH responsiveness after being cocultured for 24 h, LH was added at increasing concentrations (0, 0.01, 0.1, or 1 IU/mL) and culture continued for 48 h. Then the medium was collected for subsequent measurement.

### 2.5. Androgen Assays

The spent medium was collected at 24 h, 48 h, and 72 h. After centrifugation at 2500 ×g at 4°C for 15 minutes, the supernatant was stored at −80°C for androgen measurement. The concentrations of testosterone and androstenedione were determined using radioimmunoassay (RIA) kit (Beifang Biotechnique Institute, Beijing, China) and enzyme linked immunosorbent assay (ELISA) kit (J&L Biological, Shanghai, China), respectively, according to manufacturer's protocols.

### 2.6. Quantitative Real-Time PCR

TCs were harvested and RNA was extracted using Trizol reagents (Invitrogen, Carlsbad, CA). RNA was measured with spectrometry for OD260/280 and cDNAs were prepared with PrimeScript Reverse Transcription Kit (Takara, Dalian, China) according to manufacturer's recommendations. Real-time PCR was performed with StepOne Plus Real-Time PCR System (Applied Biosystems, USA) using SYBR Premix Ex Taq II (Takara, Dalian, China). Primers were designed for each gene using the Primer Premier 5.0 software (Premier Biosoft International, Palo Alto, CA, USA). The designation and sequences of specific PCR primers of* Star*,* Cyp11a1*,* Cyp17a1*,* Hsd3b2*, and* LHR* expression were listed in Table 1. Primers were synthesized by Invitrogen (Invitrogen Trading Co. Ltd., Shanghai, China).

Each sample was assayed in duplicate using 1 *μ*L cDNA, 10 pmol primer, and 1 *μ*L SYBR Green Master Mix (Applied Biosystems, USA) in a total volume of 20 *μ*L.* GAPDH* mRNA was measured and the results served as internal control. PCR conditions were initial denaturation at 95°C for 1 min followed by 40 cycles of amplification: denaturation at 95°C for 15 sec, annealing at 60°C for 1 min, and extension at 72°C for 3 min. Melting curve analysis was performed to confirm the specificity of amplification. The difference of CT between steroidogenic enzymes and* GAPDH* was defined as ΔCT. The difference of ΔCT between experimental and control groups was defined as ΔΔCT. The relative quantity was determined by 2^−ΔΔCT^ [17].

### 2.7. Statistical Analysis

All data were expressed as mean ± SEM from at least three independent experiments. For comparison of quantitative data of two groups, Student's *t*-test was used, and for multiple comparisons, one-way ANOVA (SPSS 16.0) was used. *P* < 0.05 was considered as reaching a statistically significant level.

## 3. Results

### 3.1. Verification on the Purity of Theca Cells

**Figure 1:**
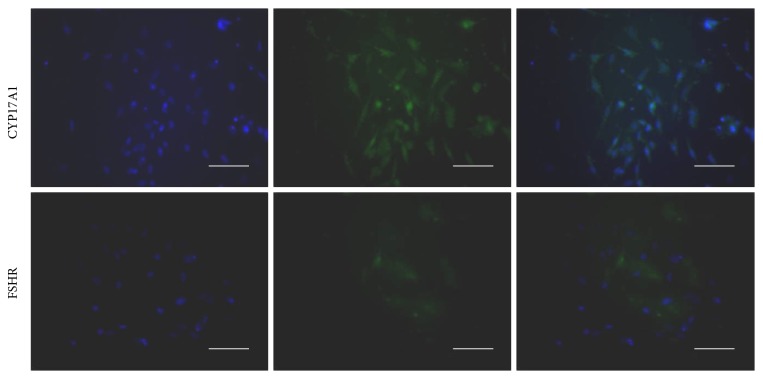
Immunofluorescence staining of CYP17A1 and FSHR in theca cells. Scale bar = 100 *μ*m. The purified cells were examined with immunofluorescence method after 24 h. By a series of titration experiments, the density of TCs was adjusted to approximately 70% confluence. FITC (green) staining CYP17A1 indicated the TCs origin. Hoechst staining (blue) indicated the location of cell nuclei. More than 90% of the cells were stained positive for CYP17A1, confirming the purity of TC cells population. Weak staining with FSHR antibody indicated that these TCs cultures were largely free of GCs contamination.

After overnight incubation, the wells coated with TCs were washed with medium to remove unattached cells. The cellular morphology and confluence of TCs were observed under the microscope. Typically, the attached cells looked thin and dendritic, indicating their TC origin. By a series of titration experiments, the density of TCs was adjusted to approximately 70% confluence. The purity of TCs was further verified with immunofluorescence staining using a TC-specific marker Cytochrome P450, family 17, subfamily A, polypeptide 1 (CYP17A1, also known as 17a-hydroxylase/17, 20-lyase), and the GC-specific marker Follicle Stimulating Hormone Receptor (FSHR) for reverse identification. The results in Figure 1 demonstrated that more than 90% of the cells were stained positive for CYP17A1, confirming these cells' TC origin. As FSHR is a GC-specific marker not TCs, we used it to confirm that the extracted cells were TCs and contain almost no GCs by reverse identification. Results from control experiments using FSHR antibody did not show significant fluorescence signal, indicating that these TCs cultures were largely free of GC contamination.

### 3.2. Effect of Granulosa Cells on the Steroidogenesis in Theca Cells

**Figure 2:**
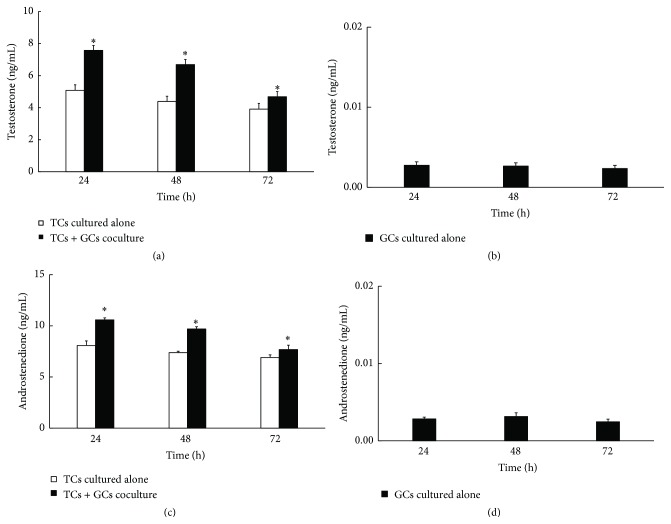
Effect of GCs on steroidogenesis in TCs. Concentrations of testosterone and androstenedione in the spent medium were measured by radioimmunoassay and enzyme linked immunosorbent assay, respectively. (a) and (c) An increased amount of testosterone and androstenedione was observed in cocultured model. (b) and (d) GCs produced diminished levels of testosterone and androstenedione, respectively. Note the scale difference between (a) and (c) as well as between (b) and (d). Data were expressed as mean ± SEM of three independent experiments. The asterisk marked the statistically significant difference.

Concentrations of testosterone and androstenedione in the spent medium were measured at 24 h, 48 h, and 72 h using RIA and ELISA, respectively. When cocultured with GCs, TCs produced an increased amount of testosterone and androstenedione than TCs cultured alone (*P* < 0.05, Figure 2(a) for testosterone and Figure 2(c) for androstenedione). Interestingly, when TCs were cultured alone, the production of testosterone and androstenedione showed a declining trend along time, which may reflect the adaptation change to* in vitro* conditions lacking the supports from other cell types. In the spent medium of GCs cultured alone, the levels of testosterone (Figure 2(b)) and androstenedione (Figure 2(d)) were both diminished in comparison with TCs. And no alterations were observed following different time of* in vitro* culture (*P* > 0.05). Taken together, these results indicated that GCs could promote TCs production of testosterone and androstenedione.

### 3.3. Expression of Steroidogenic Enzymes in the Cocultured Model

**Figure 3:**
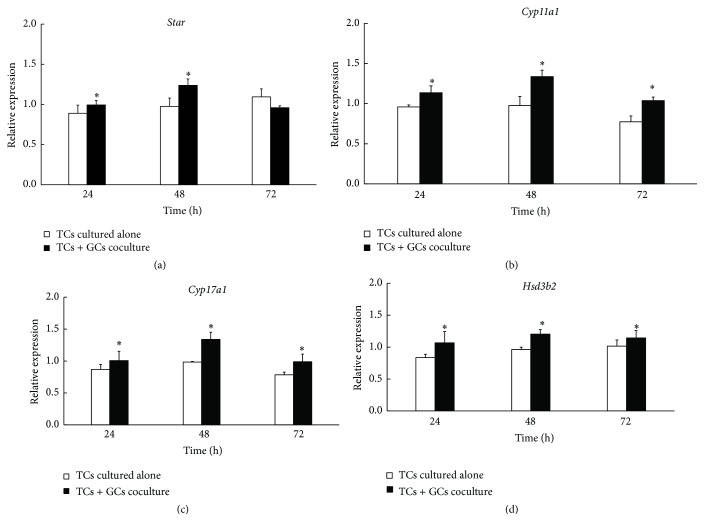
Effect of granulosa cells on mRNA expression of genes encoding steroidogenic enzymes (*Star*,* Cyp11a1*,* Cyp17a1*, and* Hsd3b2*) in TCs. After TCs were cultured up to 72 h, the expressions of* Star*,* Cyp11a1*,* Cyp17a1*, and* Hsd3b2* were measured by quantitative real-time PCR (*GAPDH* as internal control). Expressions of all these genes were increased in the cocultured model compared with TCs cultured alone. Data were the mean ± SEM of three independent experiments where all the samples were repeated in thrice. The asterisk showed the statistically significant difference. ^*∗*^ *P* < 0.05.

To investigate the regulatory pathway leading to the increased androgen production, quantitative real-time PCR was performed to determine the expression of genes involved in androgen synthesis* Star*,* Cyp11a1*,* Cyp17a1*, and* Hsd3b2* at 24 h, 48 h, and 72 h. As shown in Figure 3, the results revealed that the expressions of all these genes were increased in the cocultured model compared with TCs cultured alone (*P* < 0.05). Thus, increasing expression of the genes involved with androgen synthesis appeared to account for the increased androgen production. These results indicated that paracrine factors secreted from GCs can pass the Transwell insert membrane and regulate the transcription of steroidogenic enzymes.

### 3.4. Effects of Granulosa Cells on the Expression of* LHR* mRNA in Theca Cells

**Figure 4:**
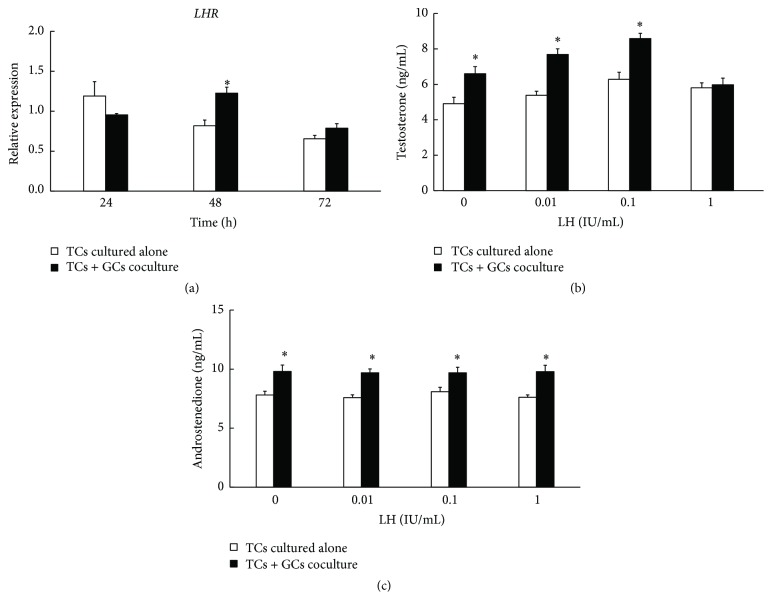
(a) Effect of granulosa cells on mRNA expression of* LHR* in TCs cells and LH stimulation of androgen production in TCs in the cocultured model. The* LHR* mRNA expression of TCs in cocultured model increased significantly at 48 h but manifested no difference compared with TCs cultured alone at 24 h and 72 h (*GAPDH* as internal control). (b) Effect of GCs on testosterone production responded to LH in TCs cells after being cocultured for 48 h. Testosterone production significantly increased in response to low concentrations of LH (0–0.1 IU/mL) in the cocultured model but no increase was observed at high concentration (1 IU/mL). (c) Effect of GCs on androstenedione production responded to LH in TCs cells after being cocultured for 48 h. Androstenedione production significantly increased in response to different concentrations of LH (0-1 IU/mL) in the cocultured model. Data were expressed as mean ± SEM of three independent experiments. The asterisk showed the statistically significant difference. ^*∗*^ *P* < 0.05.

While the* LHR* mRNA expression of TCs in the cocultured model manifested no difference compared with TCs cultured alone at 24 h (*P* > 0.05, Figure 4(a)), at 48 h the* LHR* mRNA expression of TCs increased significantly (*P* < 0.05, Figure 4(a)), indicating that paracrine factors secreted from GC cells could stimulate the* LHR* mRNA expression in TCs. The subsequent disappearance of difference between TCs cultured alone and cocultured model may reflect the suboptimal conditions related to* in vitro* environment. The cocultured GCs could enhance the capacity of LH responsiveness through increasing* LHR* mRNA expression in TCs.

It was reported that, in TCs, LH upon binding to LH receptor could regulate the expressions of a series of synthesis enzymes through activation of cAMP/PKA signaling pathway, facilitating the synthesis and secretion of steroids [18, 19]. To determine the effect of GCs on androgen production by TCs in response to LH, the levels of testosterone and androstenedione in the spent medium were measured following stimulation with LH. The results revealed that testosterone and androstenedione production significantly increased in response to different concentrations of LH in the cocultured model (*P* < 0.05, Figures 4(b) and 4(c)). However, there was no difference in the amount of testosterone (*P* > 0.05, Figure 4(b)) or androstenedione (*P* > 0.05, Figure 4(c)) even in the presence of LH when TCs were cultured alone.

## 4. Discussion

On the basis of two-cell/two-gonadotropin theory of ovarian steroid synthesis, androgen production is initiated by steroidogenic enzymes (STAR, P450Scc, P450C17, and HSD3B2) in TCs and then subsequently aromatized in GCs [20]. Based on the findings that TCs androgenic pathways can be regulated by GCs via a paracrine pathway [21], we attempted to confirm that this phenomenon exists in mouse preantral follicles using the cocultured model. In this Transwell coculture model, hormones and paracrine factors secreted from follicular cells such as steroids, growth factors, and cytokines can pass the Transwell insert membrane. The cocultured model can mimic to a certain extent the local ovarian steroidogenesis by supporting similar structural integrity of follicles. Through measuring the steroidogenesis in this model, we can observe the effects of extensive cross talk and intercellular signaling between GCs and TCs, validating the efficacy of the model for studying the cell-cell interactions. In future, the coculture can be applied to investigate the significance of interactions between GCs and TCs under various pathological conditions such as hyperandrogenemia in PCOS [22] where the expression of steroidogenic enzymes was much increased [23].

Parrott and Skinner reported that GCs could promote ovarian interstitial cells recruitment to form theca cell layers around follicles and that inhibin secreted from GCs could induce maturation of the dominant follicle and the production of androstenedione [24]. These findings indicated that GCs probably provide some essential regulating factors for TCs on steroidogenesis, such as steroids, growth factors, cytokines, and extracellular matrix and maintain the ability of TCs for androgen synthesis by paracrine regulation. In this study, we evaluated the functions of ovarian cells by measuring the change in androgen concentrations. When cocultured with GCs, TCs produced an increased amount of testosterone and androstenedione than TCs cultured alone. This increase was driven by enhancing the expressions of steroidal synthetase (STAR, P450Scc, P450C17, and HSD3B2) in TCs. As more P450Scc was expressed, cholesterol is converted to pregnenolone and progesterone. When precursor of steroid runs out, both testosterone and androstenedione productions showed a declining trend over time. This illustrates that not only oocyte-derived factors but also GCs-derived factors participate in steroid production in ovaries. How and by what pathway these autocrine/paracrine regulators derived from these follicular cell types exert effects on steroidogenesis still needs further investigation.

To determine the effect of GCs on androgen productions of TCs in response to LH, acquisition of LH responsiveness in preantral follicular TCs from the mouse ovary was evaluated. In this study, we found that the cocultured GCs could enhance the capacity of LH responsiveness on increasing* LHR* mRNA expression in TCs. The presence of GCs led to a significant increase on* LHR* mRNA expression of TCs at 48 h but manifested no difference compared with TCs cultured alone at 24 h and 72 h most likely due to the suboptimal* in vitro* culture conditions, for example, the lack of support by LH and other hormones.

It is generally accepted that LH responsiveness and responses to LH on androgen production are two well-established functions of TCs in ovary [25–27]. By cAMP/PKA signal pathway, LH could regulate the expression of a range of androgen synthetases and facilitates the transcription of* Star*,* Cyp11a1*,* Cyp17a1*, and* Hsd3b2* [28]. Previous studies have shown that the capacity of TCs on androgen synthesis and secretion in response to LH appears to be dose-dependent [5, 11, 12]. These findings are consistent with the clinical observation in women with PCOS that elevated serum LH levels positively correlate with serum testosterone concentrations [29, 30]. Rosenfield and Bordini [31] found that moderate levels of testosterone appear to stimulate LH production at both the hypothalamic and pituitary levels, while high levels of testosterone suppress the effect of LH. Another study indicated that the level of testosterone was low, while androstenedione was high in PCOS patients [32]. In our study, when treated cocultured model with different concentrations of LH (0-1 IU/mL), concentrations of testosterone and androstenedione increased in low concentrations of LH but no further increase was observed at high concentrations. These results were in accordance with previous studies, which suggested that excessive testosterone may suppress the response to LH [5, 31, 32]. Taken together, these findings indicated that GCs could strengthen the capacity of LH by facilitating the transcription of* Star*,* Cyp11a1*,* Cyp17a1*, and* Hsd3b2*, therefore inducing androgen synthesis in TCs.

As an important component of a follicle, TCs exert certain effects on steroidogenesis, follicular development, and atresia. In the present study, the* in vitro* cocultured model including GCs and TCs from mouse preantral follicles recapitulates some native ovary function. However, it is unable to completely represent* in vivo* microenvironment of whole follicles. Furthermore, it is not yet known what effects TCs-derived factors may exert on GCs and oocytes. Therefore, the function and mechanism of TCs exerted in the ovary as well as the interaction between TCs with GCs and oocytes deserve further exploration* in vivo*.

In conclusion, the present results demonstrated the key role of granulosa cells for the steroid production in theca cells. The Transwell coculture model has provided a technical basis for studying some features of interactions between follicular cell groups under different pathophysiological conditions such as that of hyperandrogenemia.

## Figures and Tables

**Table 1:** Primer sequences used for real-time PCR.

Accession number	Gene	Sequence 5′-3′	Size (bp)
NM_011485.4	*Star*	F: CCACCTGCATGGTGCTTCA	142
		R: TTGGCGAACTCTATCTGGGTCTG	

NM_019779.3	*Cyp11a1*	F: GGCACTTTGGAGTCAGTTTACAT	186
		R: GTTTAGGACGATTCGGTCTTTCTT	

NM_007809.3	*Cyp17a1*	F: TCTGGGCACTGCATCACG	124
		R: GCTCCGAAGGGCAAATAACT	

NM_153193.3	*Hsd3b2*	F: GCCCCTACTGTACTGGCTTG	212
		R: TCCCGATCCACTCTGAGGTT	

NM_001289726.1	*GAPDH*	F: AGGTTGTCTCCTGCGACTTCA	187
		R: GGGTGGTCCAGGGTTTCTTACT	

NM_013582.2	*LHR*	F: GAGACGCTTTATTCTGCCATCT	119
		R: CAGGGATTGAAAGCATCTGG	
